# An investigation of the relationship between autonomy, childbirth practices, and obstetric fistula among women in rural Lilongwe District, Malawi

**DOI:** 10.1186/s12914-017-0125-3

**Published:** 2017-06-19

**Authors:** Julika Ayla Kaplan, Jonathan Kandodo, Joseph Sclafani, Susan Raine, Jennifer Blumenthal-Barby, Alison Norris, Abigail Norris-Turner, Elly Chemey, John Michael Beckham, Zara Khan, Reginald Chunda

**Affiliations:** 1 1 Baylor Plaza, Houston, TX 77030 USA; 2 Child Legacy International, Msundwe, Malawi; 3 1841 Neil Avenue, Columbus, OH 43210 USA; 4 6100 Main Street, Houston, TX 77005 USA; 5 Malawi College of Medicine, Blantyre, Malawi

**Keywords:** Malawi, Obstetric fistula, Autonomy, Women

## Abstract

**Background:**

Obstetric fistula is a childbirth injury caused by prolonged obstructed labor that results in destruction of the tissue wall between the vagina and bladder. Although obstetric fistula is directly caused by prolonged obstructed labor, many other factors indirectly increase fistula risk. Some research suggests that many women in rural Malawi have limited autonomy and decision-making power in their households. We hypothesize that women’s limited autonomy may play a role in reinforcing childbirth practices that increase the risk of obstetric fistula in this setting by hindering access to emergency care and further prolonging obstructed labor.

**Methods:**

A medical student at Baylor College of Medicine partnered with a Malawian research assistant in July 2015 to conduct in-depth qualitative interviews in Chichewa with 25 women living within the McGuire Wellness Centre’s catchment area (rural Central Lilongwe District) who had received obstetric fistula repair surgery.

**Results:**

This study assessed whether women’s limited autonomy in rural Malawi reinforces childbearing practices that increase risk of obstetric fistula. We considered four dimensions of autonomy: sexual and reproductive decision-making, decision-making related to healthcare utilization, freedom of movement, and discretion over earned income. We found that participants had limited autonomy in these domains. For example, many women felt pressured by their husbands, families, and communities to become pregnant within three months of marriage; women often needed to seek permission from their husbands before leaving their homes to visit the clinic; and women were frequently prevented from delivering at the hospital by older women in the community.

**Conclusions:**

Many of the obstetric fistula patients in our sample had limited autonomy in several or all of the aforementioned domains, and their limited autonomy often led both directly and indirectly to an increased risk of prolonged labor and fistula. Reducing the prevalence of fistula in Malawi requires a broad understanding of the causes of fistula, so we recommend that the relationship between women’s autonomy and fistula risk undergo further investigation.

**Electronic supplementary material:**

The online version of this article (doi:10.1186/s12914-017-0125-3) contains supplementary material, which is available to authorized users.

## Background

Obstetric vesicovaginal fistula is a childbirth injury caused by prolonged obstructed labor that results in tissue destruction. This process can create a hole in the tissue wall between the vagina and bladder, which may cause victims of obstetric fistula to experience chronic urinary incontinence. Obstetric fistula is preventable and treatable, but conservative estimates indicate that 1.6 out of every 1000 Malawian women are affected by the condition [7]. Although obstetric fistula is directly caused by prolonged obstructed labor, many other factors indirectly increase the risk of obstetric fistula among pregnant women in rural Malawi. For example, unskilled birth attendants may not immediately recognize the need for specialized obstetric care; women who become pregnant when their pelvises are still physically immature may struggle during labor and delivery; and pregnant women in rural areas may have limited access to emergency obstetric care due to poverty, poor transportation, and distance from health facilities. Scholarship from Malawi indicates that many women in rural Malawi have limited autonomy and decision-making power in their households [11, 13], which raises the question whether gender inequality plays a role in reinforcing childbirth practices that increase the risk of obstetric fistula in this setting. Therefore, the indirect factors that increase the risk of obstetric fistula in rural Malawi warrant further investigation.

Reducing the worldwide prevalence of obstetric fistula requires improving access to high-quality healthcare and emergency obstetric services in resource-poor regions. However, even when women receive vouchers for transportation to the hospital, free access to high-quality medical care, and extensive reproductive health education, many still deliver in their homes without skilled birth attendants present [11]. This suggests that factors in addition to poverty and geographic isolation—such as women’s limited autonomy and decision-making power—may impact their ability to seek reproductive healthcare. Women’s autonomy has been defined as “the degree of women’s access to, and control over, material and social resources within the family, community, and society at large” [5]. More specifically, Upadhyay et al. [12] define *reproductive* autonomy as a woman’s ability to make decisions about contraceptive use, pregnancy, and childbearing. When women lack reproductive autonomy, they may be unable to discuss their reproductive health with their partners and family members, which can lead to lower utilization of maternal health services and poor health outcomes for both mothers and children [1].

Although many studies have demonstrated that gender inequality affects women’s ability to negotiate reproductive health practices [3, 6, 8], few studies have investigated the role that gender inequality plays in women’s negotiation around childbirth practices, particularly around practices linked to obstetric fistula. Many women in sub-Saharan Africa deliver at home with the help of relatives or traditional birth attendants and require permission from their husbands or mothers-in-law to seek medical care [10]. If women lack access to financial resources and are not active decision-makers regarding contraception, access to antenatal care, and healthcare utilization during labor and delivery, their limited autonomy might reinforce childbearing practices that increase their risk of developing obstetric fistula.

This study assessed whether gender inequality in rural Malawi reinforces childbearing practices that increase women’s risk of obstetric fistula by considering four dimensions of autonomy: sexual and reproductive decision-making, decision-making related to healthcare utilization, freedom of movement, and discretion over earned income. These four dimensions were developed based on an article called “Women’s autonomy in rural India: Its dimensions, determinants, and the influence of the context” [4] that identified economic and child-related decision-making, mobility, freedom from threat from husband/partner, access to economic/social resources, and control over economic resources as indicators of autonomy that influence women’s health outcomes. By exploring these four dimensions, this study examined whether women in rural Malawi are able to decide when to become pregnant, choose whether to seek healthcare before and during delivery, travel freely to health facilities, and spend family income on healthcare-related expenses, all of which may affect their risk of fistula development.

## Methods

In order to conduct this study, the principal investigator (JAK) partnered with a Malawian researcher (JK) to conduct in-depth qualitative interviews in Chichewa with 25 adult women who had experienced obstetric fistula (see Additional file 1). Participants were recruited from the rural catchment area surrounding the McGuire Wellness Centre in Msundwe, Malawi, which contains nearly 18,000 people living in 68 villages, most of whom work in agriculture. JK identified eligible patients by advertising the study to community leaders, who then told their community members about the opportunity to participate in the study.

After the participants presented at the McGuire Wellness Centre, agreed to participate in the study, and gave informed consent, the researcher spent approximately one hour conducting and audio recording semi-structured interviews in Chichewa privately with the participants. The following demographic information was collected on each participant at the beginning of the interview: village, current age (youngest: 25, oldest: 61, average: 41), age at marriage (average: 19), level of education (8 had no formal education), distance to closest health facility, current marital status, marital status after fistula, and relative socioeconomic status (17 “low,” 8 “medium”), which was determined by JK based on his experience working with the population.

After data collection at the McGuire Wellness Centre in Malawi was complete, JAK brought the data back to Baylor College of Medicine, where she conducted qualitative content analysis with JBB, JMB, and ZK. They used the grounded theory method, which involves inductively identifying themes that are “grounded in” or based on recurring statements in data from participants, to identify the role different dimensions of autonomy may have played in the labor and delivery process [2]. Interview transcripts were uploaded and analyzed using ATLAS.ti (http://www.atlasti.com/), a computer-assisted qualitative data analysis (CAQDAS) program that provides a platform to analyze interview transcripts.

Their analytical process involved developing a series of codes collaboratively through discussions between JAK, JBB, JMB, and ZK. These codes were developed with the intention of identifying participant quotations that were related to the four domains considered in this study (sexual and reproductive decision-making, decision-making related to healthcare utilization, freedom of movement, and discretion over earned income). Code assignments to preliminary transcripts were made independently by members of the research team and later compared and discussed until a coding consensus was reached. This process of analysis allowed the research team to calculate the frequency with which each dimension of autonomy was addressed throughout the interview process.

## Results

### Domain I: Sexual and reproductive decision making

In this section, we examine whether the women we interviewed in rural Malawi typically decided when to become pregnant, when to have sex, and whether to use contraception, and whether those factors affected their risk of developing obstetric fistula during labor or delivery.

#### Pregnancy

We found the theme of diminished autonomy relating to pregnancy in 16 of our interviews (see Table 1). Many of the women we interviewed said they felt pressured by their husbands, families, and communities to marry and get pregnant at a young age, sometimes before they were physically mature enough to bear children. When we asked one participant why she got married at a young age, she said, “I just decided to get married since there was nothing I was doing at home.” The average age of marriage among our participants was 19, with the youngest at age 13, which indicates that many became pregnant before achieving physical maturity. One participant said, “I told [my husband] that my wish [was] to get pregnant after a year, and he said that he would not accept that; he wanted me to get pregnant immediately, and there was nothing I could do.”

**Table 1:**
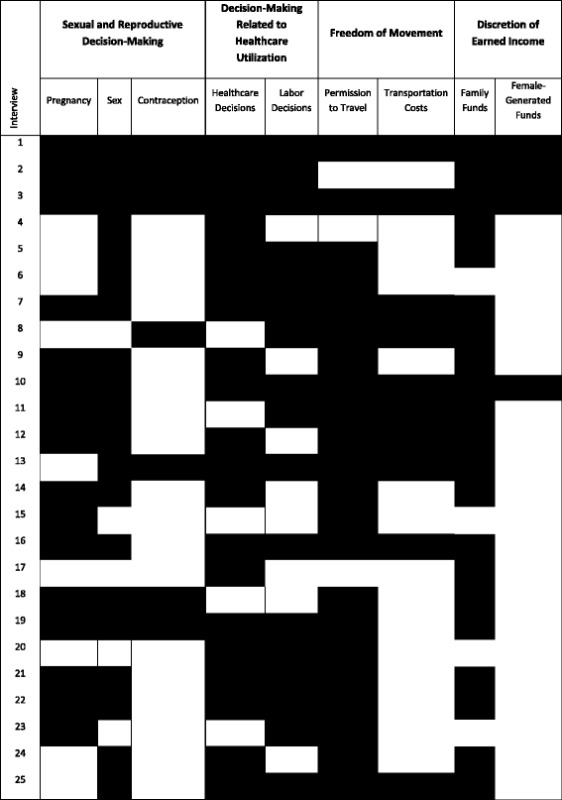
Visualization of autonomy across all domains for all interviews

This table is based on the ethnoarrays designed by Corey M. Abramson and Daniel Dohan in the article “Beyond Text: Using Arrays to Represent and Analyze Ethnographic Data” published in *Sociological Methodology* in 2015. In the table, a black box represents limited autonomy for a particular participant in a given domain

The participants explained that men earn respect in the community by fathering children soon after marriage to prove their fertility. In fact, many men threaten to divorce their wives if they do not bear children soon after marriage because “there is no way a man can keep a woman who is not bearing children, no matter how beautiful and faithful that woman is.” Family members often expect newly married couples to bear children soon after marriage for the same reasons, even if they do not have the resources to support them: “parents always expect you to have children within the first year of marriage, just as a sign of prestige… even when you don’t have anything you can give to the children.” In fact, when one participant was not pregnant within a year of marriage, “people were saying that [she] had *masungu* (vaginal sores).” In several cases, this pressure did not disappear after a single pregnancy; for example, one participant said, “People had already started calling our family names and saying that my husband [was] failing to do his job… that is why when I had the first child, it did not take us long to have another baby… my husband said he wanted people to know he [was] capable of doing the man’s job.”

In many cases, participants explicitly stated that they would have preferred to wait several years after marriage before bearing children, either so they could accumulate more resources to support the children or because they felt their bodies were not mature enough to bear children yet. Despite these desires, many of these participants became pregnant soon after marriage in response to the pressure they were experiencing. One participant said, “I was thinking that I [would] wait for some years before getting married, but everybody was encouraging me to get married even though I was young.” Some of these women even expressed a clear understanding of the link between early pregnancy and increased risk of obstetric fistula. One participant said, “I wanted my body to be mature so I would not have delivery complications,” and another said, “I was thinking I would wait for three to four years before getting pregnant, but my parents-in-law said they wanted… children, and that is one of the reasons why I had fistula.”

#### Sexual intercourse

We found the theme of diminished sexual autonomy in 20 interviews (see Table 1). Almost all of the women we interviewed said they felt pressured to have sex with their husbands, both immediately after marriage and throughout their marriages. The participants described several different ways in which men used their status as the “heads of the household” to pressure women to have sex before they were ready: men whose wives refused to have sex complained to the community marriage counselors (experienced women who mediate marriage-related disputes) that their wives were not fulfilling their duties, refused to speak to their wives, refused to buy their wives new clothes, accused their wives of having affairs, or threatened to find another partner who was willing to have sex. For example, one participant said, “I had been punished in the past for refusing to have sex, and it was one of the worst experiences [of my life] because my husband said that if I refused [to have sex], he would marry another woman.” Both men and women expressed the belief that sex is the reason for marriage and that women have a duty to satisfy the “sexual needs” of their husbands. In fact, one participant said, “There will be problems [if I refuse to have sex]; the man will say if he had married for the sake of having someone to cook for him or wash clothes for him, he would have stayed at his mother’s house.”

Many of the women we interviewed described their first sexual encounters with their husbands as instances of unwanted sexual intercourse. Several felt completely ignorant about the mechanics of sex and their husbands’ expectations before their wedding nights. One participant reported that the “elderly women” in the village taught her during her pre-marital years that “when you have sex with men, your stomach will swell and you will die.” Another participant said, “I was forced [to have sex]. I had refused, but he pulled me down and came on top of me. Do you call that sex or war?” Finally, one participant said, “On the fourth night [of my marriage], my husband… pulled off my underpants… I accepted it, but it was a terrible experience and I did not sleep the whole night.” A woman who was “crying and screaming” during her first sexual encounter with her husband developed genital wounds that took weeks to heal. She went back to her parents and told them she wanted a divorce, but her parents told her she had to go back to her husband. Even after years of marriage, many of the women we interviewed associated sex with duty, not pleasure; they “became used to [sex]” or “came to understand that [sex] is something that happens in the family,” even if they “never had any desire to have sex.” Some participants even felt that they had developed fistulas because their experiences with unwanted sexual intercourse had directly led to pregnancy before their bodies were mature enough to bear children: “this very same forceful sexual act resulted in pregnancy, and it was disturbing for me because I did not enjoy it at that time and was not ready for pregnancy.”

We asked the women we interviewed whether they believed their husbands had the right to get angry when they refused to have sex. Although some participants said their husbands did have the right to get angry because “they had refused to listen” or “because the men are the heads of the family,” many women said they did not believe men had the right to demand sex from their wives. One woman said, “No, why should violence be justified? We suffer a lot because we have no voice.” Another woman said, “No, I do not think he has the right to be angry or punish the woman for refusing to have sex, but I am the one who is being *kundipanikiza* (pressured) to do things that I do not want to do. It is me whose *ufulu waphwanyidwa* (rights have been violated).” However, despite these beliefs, many of these women still had sex with their husbands because they feared the threat of divorce or did not feel comfortable discussing sex.

#### Contraception

We found the theme of diminished autonomy relating to contraception in 7 interviews (see Table 1). We discovered many reasons why participants did not use contraception, which would have given them the autonomy to decide when to became pregnant. In some cases, their parents discouraged them from using contraception, often because they held false beliefs about the efficacy or safety of contraception. For example, one woman said, “my parents discouraged me from using [an IUD], saying it would harm me, so I decided not to use any contraception.” Other participants had the desire to use contraception, but were misinformed about the efficacy of various methods: “We tried *yachikuda* (traditional method of family planning), but it did not help. I took all the herbal medicines, but… I still got pregnant.” Some of the women we interviewed said they were afraid to discuss contraception with their husbands because they believed their husbands would accuse them of having affairs or divorce them when they were no longer interested in bearing children. Some participants believed that men were responsible for initiating conversations about contraception and deciding the sizes of their families because they have a better understanding of the family’s resources. Finally, some women never discussed contraception with their husbands because they wanted to leave family planning in the hands of God.

Despite the general belief that men were responsible for making decisions related to contraception, several of the women we interviewed still demonstrated their autonomy by seeking contraception without consulting their husbands. When women knew their husbands would not support the use of contraception, many “just went to the clinic and got the injection (Depo-Provera).” When we asked these participants why they chose Depo-Provera, a method of birth control that only requires one injection every three months, they said, “You can use it without the knowledge of your husband, unlike the pills, which you have to take daily at home.” Because of this, they believed Depo-Provera was “safe for most women” who feared retribution from their husbands. This suggests that, even though some women decided to use contraception without their husbands’ permission, they were still forced to make certain decisions, such as which type of contraception to use, by considering which option would be least likely to upset their husbands.

### Domain II: Decisions related to healthcare

In this section, we consider whether the women we interviewed had the ability to choose whether to seek healthcare before and during delivery and whether this influenced their risk of developing obstetric fistula.

#### Decisions about healthcare utilization

We found that 20 of the 25 participants experienced diminished autonomy when making decisions about healthcare utilization (see Table 1). Our participants listed many reasons why their husbands were typically responsible for making healthcare decisions, some of which related to the other domains identified in this paper. For example, some women explained that male decision-making power was directly related to control over money and other household resources. In one case, a participant’s husband made the decision whether his wife would attend her antenatal clinic appointments because “he was the one who took her to the hospital on the family bicycle” or made other travel arrangements. Another woman stated that “the people who have money are the ones who can make decisions”; in most cases, this translated to male decision-making power because, as one participant said, “We are poor as women.”

Other women gave cultural explanations for their limited decision-making power, stating that men make healthcare decisions because they are the “heads of the family,” and there are “some rules you have to follow, otherwise you will spoil your marriage.” Another participant said her husband “is like the leader of the house, and nothing can be done without his consent.” Finally, one woman suggested that it would be “rude” and disrespectful towards her husband if she made a decision about her health without her husband’s input.

Several women described themselves as being “looked down upon as children of the family” or described their husbands as “parents.” In many cases, this was related both to the young age of the wife and to whether the wife was living *chitengwa*. *Chitengwa* is a practice believed to take place in most of Malawian society in which, upon marriage, a woman leaves her village to live in her husband’s home village [9]. According to the Malawi Human Rights Commission, patrilineal societies practice *chitengwa* “to confirm that the woman belongs to the man’s side after marriage” and “to signify that the man is in full control of the woman.” Because of this, while living *chitengwa*, women are often subordinate to both their husbands and their mothers-in-law. In one case, a young woman lived in her parents’ home, which gave her parents the authority to make healthcare decisions for her. Another participant proposed a link between decision-making power and level of education, claiming that she was “like a child to [her] husband” because “[he thought she didn’t] have any wisdom… since [she] did not go to school.” One woman agreed with this belief and preferred to let her husband make healthcare decisions on her behalf: “he has been to school so he says he knows more things than I do.”

Despite this example, the vast majority of participants expressed a desire to make their own healthcare decisions. In one case, a participant’s husband forbade her from visiting the antenatal clinic because he only believed in traditional medicine; however, the participant demonstrated her autonomy by traveling to a clinic far from her house without her husband’s knowledge even though she said she would be beaten if caught. Some women believed they deserved to have decision-making power because they understood their healthcare needs better than their husbands did: “if you are sick, the people around you might not understand the severity of your sickness and cannot tackle the problem the way you could if you handled it yourself.” In addition, several participants felt that their husbands did not prioritize women’s health and might accuse them of laziness if they asked to spend the day at the antenatal clinic instead of working at home. Finally, one woman said her husband would wonder “why [she has] to waste time going to the antenatal clinic when [she has] had safe deliveries at home” in the past.

#### Decisions about labor and delivery

We found diminished autonomy related to labor and delivery decision making in 17 of our interviews (see Table 1). When we asked participants about decision-making during the labor and delivery period, many gave stories of limited autonomy leading to prolonged labor in the absence of adequate medical care, which is a direct cause of obstetric fistula.

Our interviews indicated that final authority over labor and delivery decision-making ultimately resided with the husband. However, men often deferred to the advice of the “elderly women” in the village because the men “had no experience with childbirth and pregnancy.” The elderly women often included female relatives, *azambas* (traditional birth attendants who provide basic care and support during childbirth in remote areas based on knowledge and experience acquired through informal education and training), and other respected women in the village. In several cases, the opinions of the elderly woman received priority over those of the doctors providing antenatal care. For example, one participant had a history of complicated deliveries and another was told at the antenatal clinic that she needed to “deliver at the hospital,” but in both cases, the husbands refused and “[were] just listening to everything the elderly women were telling [them].” In some cases, husbands and family members might have had other motives for refusing hospital deliveries: two women accused their husbands of wanting them to die in childbirth, and one mother-in-law delayed transport to the hospital in an attempt to prove that her daughter-in-law had been promiscuous.

There was a near-universal desire among the women we interviewed to deliver at the hospital, but in most cases, this desire was refused until the situation was determined to be beyond the ability of a *mzamba*, or traditional birth attendant. One husband told his wife that she “should not be troubling people as if it were [her] first time to deliver at home…when all along [she has] had safe deliveries at home through the help of a *mzamba*.” Even when women were pregnant for the first time, men asked their wives, “Why should you bother going to the hospital when all the women here deliver at home safely?” Some men used their control over the family finances to enforce their decisions, stating that they would not support the expenses of a hospital delivery, while others simply relied on their authority as the leaders of the family. Even after it became apparent that women were experiencing complicated labor, they often continued to labor in distress for 1–3 days before they were finally taken to the hospital. This was occasionally due to indecision among the elderly women and their husbands over whether the *mzamba* was still capable of delivering the child, but also occurred when the husband or female relatives were not present to authorize transport.

One attempt to avoid these delays is a Malawian practice known as *chidikiliro*, in which women, particularly those whose pregnancies are considered high-risk, are advised to go to a maternity waiting home near the hospital one month before their due dates to wait for labor to begin. As described above, some men refused to allow a hospital delivery at all. In other cases, however, men agreed to support a hospital delivery, but asked their wives to delay *chidikiliro* for various practical, financial, and familial reasons, which was problematic on several occasions. Many of our participants said their husbands were the only people who could formally authorize transport to the hospital. If their husbands were not present when labor began or did not leave sufficient funds to pay for transport to the hospital, then the women were left at home in distressed labor for days while they awaited their husbands’ return.

In some cases, it was difficult to determine whether husbands refused to allow *chidikiliro* or a hospital delivery for legitimate financial reasons. It is well-established that poverty constrains healthcare decision-making, but we believe that constraints on female autonomy constitute an additional obstacle. The fact that many of these women did ultimately end up delivering in hospitals after prolonged labor suggests that real financial obstacles could be navigated if they were sufficiently prioritized. Therefore, these financial constraints reinforce the important role that lack of female control over family funds plays in the ultimate development of obstetric fistula.

### Domain III: Freedom of movement

In this section, we consider whether the women we interviewed were able to travel freely and independently to healthcare facilities and whether this affected their risk of developing obstetric fistula.

#### Permission to travel

We found that 22 of the women we interviewed needed to seek permission from their husbands before leaving their homes (see Table 1). In many cases, the husbands granted permission for their wives to travel (e.g., to the clinic), but the women still needed to inform their husbands and get permission first. Many women said there was “no way” they could leave without telling their husbands, and they described the underlying reason as the need to show respect and avoid *kusamvera mamuna* (being insubordinate towards their husbands) or *kuwadelera* (overlooking or belittling their husbands). As one woman said, “…he is the head of the house and you cannot just leave your home without informing the husband that you want to go out… so whenever I travel, I make sure he knows about it and has agreed to what I want to do and where I want to go.” Several women indicated that a man might refuse to allow his wife to travel if he believes she is having an extramarital affair or making an excuse to avoid work or other chores. In addition, a man might prevent his wife from traveling as a form of punishment after a quarrel: “If you have had a quarrel with your husband, obviously he will not allow you to leave the house [as] a form of punishment.”

#### Transportation costs

We found the theme of diminished autonomy relating to transportation costs in 10 interviews (see Table 1). When women do have not control over the household income and therefore cannot afford transportation to the hospital, they are unable to travel freely to meet their healthcare needs. This barrier was not significant in the case of antenatal check-ups because women could (and often did) walk together in groups to the clinic. It was, however, occasionally a barrier during delivery due to the need for money to pay for bike taxis or vehicles. One woman said, “[My husband] told me that [if] I wanted to deliver at the hospital, I should find my own means of traveling there because he would not be able to support me.” Another participant said, “When [my husband] came back, he was sad because he had not left me with enough money… if he had, people would have hired a vehicle to take me to the hospital; this is why I was saying that I need to be involved in making decisions.”

### Domain IV: Discretion over earned income

Finally, in this section, we consider whether the women we interviewed were able to spend family income and independently generated income on healthcare-related expenses.

#### Family funds

Issues pertaining to women’s control over family funds appeared in 21 of 25 interviews (see Table 1). When women have at least partial discretion over family income, they have an increased ability to spend money on healthcare, including hospital fees and transportation to antenatal clinics. It was relatively common in our interviews, however, for men to have total control over both the family income (income primarily from harvesting, to which the entire family contributes) and their own personal income (the pay they receive for independent work).

Most of the women we interviewed said their husbands control the family income because “*wamkuru wabanja ndi mamuna* (they are the heads of the household).” Because of this, there is an expectation that women will respect their husbands’ financial decisions without disagreement. To explain this point, one woman said, “The man is the head of the family, and you cannot have two heads in a family.” Several participants said they could ask their husbands for money as long as they did not appear to be challenging or undermining their authority, which might lead them to withhold money or stay with other wives. One participant concluded that “for the sake of peace in the family, [she] should let [her husband] make decisions about the money he earns.”

Despite their limited role in making decisions about family finances, many of the women we interviewed believed that women are capable of spending money more wisely than men because, they claimed, unlike men, they take the needs of the whole family into account. In almost every case, participants expressed a desire to contribute to decisions about how the family income should be spent. One woman said, “Do you think a man can listen to you when there is money in his pocket? No… *osauka alibe mau* (when you are poor, you don’t have a voice).”

#### Female-generated funds

We found that 4 of the women we interviewed also had limited control over money they earned themselves (see Table 1). To supplement the money generated by the family, roughly half of the women we interviewed did piecework such as growing crops in the wetlands, drawing water for brick-molding, and working in people’s gardens. In some cases, women worked because they could not depend on their husbands for money, either because their husbands were polygamous and away from the household for long periods of time, mismanaged family funds, or withheld money from their wives as a form of punishment. One participant said, “I sometimes do *ganyu* (piecework) to supplement the family’s income because my husband is a polygamous person. His resources cannot meet the needs of all the families he has.”

In most cases, women were able to keep the money they earned themselves; however, men occasionally demanded access to this money, either outright or by refusing to buy household essentials and putting this burden on their wives. Therefore, some participants reported hiding the money they earned from their husbands. For example, one woman said, “[My husband] wants to use the money immediately. He promises that he will pay it back, but he ends up not giving back, so it is like he is robbing you.”

## Conclusion

In summary, the women we interviewed experienced limited autonomy in many different areas, including sexual and reproductive decision making, decision making related to healthcare utilization, freedom of movement, and discretion over earned income, all of which could lead to increased risk of obstetric fistula and other harms. For example, we found that many women experienced pressure from their husbands, families, and communities to become pregnant, that women often experienced unwanted sexual intercourse soon after marriage, and that women were often discouraged from using contraception; in many cases, these factors caused women to give birth before their bodies were physically mature. We also found that women’s husbands and mothers-in-law often made decisions about healthcare access, sometimes even insisting that women deliver in their communities instead of at the hospital because they had done so successfully in the past; and that women often delivered without trained healthcare professionals. In addition, our interviews indicated that women were often unable to leave their homes and travel to the hospital without asking for permission, and because they did not have control over household funds, were unable to make independent decisions about travel; this conditional healthcare access increased the risk that women would deliver in their communities or experience prolonged labor before transport to the hospital. Finally, we found that women were very unlikely to have control over family funds or, on occasion, funds they had generated themselves, limiting their ability to spend money on medical care during pregnancy. Based on these findings, we conclude that our participants’ limited autonomy in multiple areas often led both directly and indirectly to an increased risk of obstetric fistula, and we believe that health outcomes may improve if these factors can be mitigated.^1^

## Discussion

Four specific findings that merit further discussion emerged from our interviews. First, our interviews suggested that there was a relationship between the value women were perceived to hold in the community and their risk of developing obstetric fistula. For example, several of the women we interviewed married at a young age because they had dropped out of school and their mothers or grandmothers told them there was no reason for them to live in the house anymore. Because these women were valued for their role as wives, they were encouraged to marry before they were mature enough to deliver a baby. Continued changes to improve girls’ access to schooling through secondary education will permit more girls, especially girls in underprivileged areas, to stay in school and reach their full potential.

Another trend we identified in our interviews was the way in which limited female autonomy resulted in traumatic first sexual encounters for many women. In some cases, women were entirely unaware of sexual mechanics and expectations; however, in one arranged marriage where sexual “orders” on the wedding night were made very clear by those arranging the marriage, the woman at least knew that in giving her consent to marriage, she was also giving consent to sex, and she was not among those who described her first night traumatically. Encouraging this pragmatic approach might diminish the trauma that many of our participants experienced; however, this recommendation reinforces the expectation that sex is a female duty to her husband that cannot be refused. Another potential suggestion might be found in the example of one participant who rejected her husband’s right to punish her for refusing sex because she was educated by her church: she said, “our bodies are not the property of our husbands… we have control over them on how and who should use them.” A similar message was conveyed by a participant who heard “on the radio that women have rights and should be respected and honored by [their] husbands.” These examples suggest that institutions such as churches and radio that have authority and wide penetrance in Malawi might be an effective tool for promoting behavioral interventions.^2^

We also found that, despite experiencing significant impairments in the exercise of autonomy with respect to health-related decisions and behaviors, women had a desire for more autonomy and sometimes found subversive ways to express their autonomy. The most common example involved the secret use of contraception; many women sought contraception without informing their husbands. Some women indicated that they believed it was “their job” to initiate conversations about contraception, but fewer actually did initiate these conversations or serve as decision makers. Research is needed for interventions that might help close the gap between beliefs, intentions, and actual behaviors.

Finally, we draw attention to the role some women played in restricting participants’ autonomy during labor and delivery. Many of the women we interviewed wanted to deliver in a hospital setting, feeling that they would receive better care there, but were forced to deliver at home with a *mzamba*. In many cases, the person restricting the autonomy of the pregnant woman was another woman or group of women, which usually included the woman’s mother-in-law and elderly female members of the village. Educating these key community figures about delivery risks and encouraging them to advocate for pregnant women could help reduce the incidence of obstetric fistula. Further studies should research how interventions intended to promote women’s solidarity impact reproductive health and autonomy.

### Study limitations

We faced several limitations in this study including small sample size limited to a specific geographic region and recall bias. Due to time constraints, we were only able to interview 25 women who had experienced obstetric fistula. Although we did feel that our interviews reached saturation, our results might have contained more depth if we had been able to interview more women. We were also only able to interview women with obstetric fistula in the communities surrounding the McGuire Wellness Centre, and this community is not necessarily representative of all of rural Malawi. In addition, some of the participants we interviewed were in their forties and fifties, so they were recalling experiences that had occurred over twenty years ago; therefore, their memories may be subject to recall bias. Finally, as outside observers, we are aware of the risk that this study could veer into a cultural criticism for which we are ill-equipped and disinclined, so our goal has simply been to give voice to the desire for autonomy expressed by many of the women we interviewed, one of whom said, “Thank you for coming to my house today. This is a rare opportunity to tell our stories.”

## Additional file

Additional file 1: This interview guide was used during interviews with study participants. (DOC 41 kb)

## Footnotes

1 We do not intend to diminish the crucial relevance of socioeconomic factors and suggest that health promotion interventions that target women’s autonomy alone would be sufficient to drastically decrease obstetric fistula rates.

2 We would like to acknowledge that marital expectations and obligations are complicated in many different cultural contexts; this observation is not specific to Malawian culture.
