# Supplementary material for: An investigation of the relationship between autonomy, childbirth practices, and obstetric fistula among women in rural Lilongwe District, Malawi
Source: BMC Int Health Hum Rights. 2017 Jun 19;17:17. doi: 10.1186/s12914-017-0125-3 (PMC5477240; doi:10.1186/s12914-017-0125-3)
Supplement: Additional file 1: — This interview guide was used during interviews with study participants. (DOC 41 kb) [file 12914_2017_125_MOESM1_ESM.doc]

**Additional file 1**

**Dimension of Autonomy I: Sexual and Reproductive Decision-Making**

Goal: To determine the extent of the participant’s involvement in decisions relating to sexual relations and reproduction

Control over **sexual relations**

- How old were you when you first had sex, even if it was before marriage or it was forced? / *Munali ndi zaka zingati pamene munayamba zogonana ndi mamuna, ngakhale kuti munali musanakwatiwe kapena kuti anachita kukukakamizani?*
- Was your first sexual encounter forced on you, or did you agree to have sex at that time? / *Kodi kugonana kwanu koyamba anakukakamizani kapena munachita kugwirizana kuti mugonane panthawi imeneyo?*
- If you refused to have sex with your partner, would he have the right to be angry or reprimand you? / *Mutakana kugonana ndi wokondedwa wanu, kodi ali ndi ufulu wokwiya kapena wokukalipirani?*
- How easy is it for you to discuss when to have sex with your partner? / *Kodi ndikophweka bwanji kuti inu ndi wokondedwa wanu mukambirane za nthawi yoti mugonane?*
- Do you feel pressure from anybody else (such as your mother-in-law) to have a sexual relationship with your husband? / *Kodi mumamva kukakamizidwa kuchokera kwa wina aliyense (monga ngati apongozi anu) kuti mugonane ndi mwamuna wanu?*

Control over **reproduction (contraception and pregnancy)**

- Did you use contraception before your first pregnancy? / *Mudagwiritsapo ntchito kulera musanakhale ndi mimba yanu yoyamba?*
- Which family planning methods have you (or your partner) ever used? / *Ndi njira ziti zomwe inu (kapena amuna/akazi anu) mwagwiritsapo ntchito?*
- Have you and your partner ever discussed the number of children you would like to have? / *Kodi munayamba mwakambiranapo ndi amuna anu pa nambala ya ana omwe mumafuna mutakhala nawo?*
- Who usually decides whether or not you should use something to keep you from getting pregnant? You, your partner, you and your partner jointly, or someone else? / *Kodi kawirikawiri ndi ndani amene amachita maganizo oti mugwiritse ntchito njira ina yake kuti musakhale ndi pakati? Inu, amuna anu, inu ndi amuna anu limodzi kapena munthu wina?*
- Who initiates conversations about family planning? You, your partner, you and your partner jointly, or someone else? / *Kodi ndi ndani amene amayambitsa nkhani za kulera? Inu, amuna anu, inu ndi amuna anu limodzi kapena munthu wina?*

**Dimension of Autonomy II: Decision-Making Related to Healthcare**

Goal: To determine the extent of the participant’s involvement in decisions relating to access to healthcare

Control over **access to healthcare related to pregnancy and birth**

- Who usually makes decisions about health care for yourself: you, your partner, you and your partner jointly, or someone else? / *Kodi kawirikawiri ndi ndani amene amapanga maganizo opita kuchipatala inu mukadwala: inu, amuna anu, inu ndi amuna anu limodzi kapena munthu wina?*
- How many visits in total did you make to any health facility during your last pregnancy, not including the delivery? / *Pa nthawi yomwe munali oyembekezera kodi munapitako kangati ku chipatala/sikelo, kupatula ulendo omwe munapita kokachira?*
- Some people deliver at home, others in a clinic. Where did you deliver? / *Anthu ena amachilira kunyumba, ena kuchipatala. Kodi inuyo munachilira kuti?*
  - Why didn’t you deliver at the hospital? *Nchifukwa chiyani simudakachilire kuchipatala?*
- When you decided where to deliver your baby, who made the decision? You alone, your partner by himself, or you and your partner together? / *Pamene mudaganiza za komwe muzakaberekere mwana wanu, kodi ndi ndani amene adachita chiganizochi? Inu panokha, amuna anu paokha, inu ndi amuna anu limodzi?*
- When you were experiencing obstructed labor, did you go to a health facility immediately? / *Pamene munavutika kuti mubadwitse mwana, kodi munathamangira kuchipatala?*
  - If so, did anybody disagree with your decision to go to a health facility immediately? / *Ngati ndi choncho kodi panali munthu wina amene adatsutsana ndi maganizo anu ofuna kuthamangira kuchiptala?*

**Dimension of Autonomy III: Freedom of Movement**

Goal: To determine the extent of the participant’s involvement in decisions relating to access to transportation

Control over **access to transportation related to pregnancy and birth**

- How far do you live from your nearest health facility? How do you travel there? / *Kodi pali mtunda wautali bwanji kuchoka kumene mumakhala kukafika kuchipatala chanu chapafupi? Kodi mumayenda bwanji kuti mukafike kuchipatalako?*
- How long does it take for you to travel to your nearest health facility? Does it cost money for you to travel there? / *Kodi zimakutengerani nthawi yayitali bwanji kuti mukafike kuchipatala chomwe muli nacho pafupi? Kodi pamafunika ndalama ya thranspoti yoti muyendere kukafika kumeneko?*
- Are you able to travel to a health facility without the company of another adult? / *Kodi mumatha kupita nokha kuchipatala popanda kufuna munthu wina kukuperekezani?*
- When you want to visit a health facility, do you need to ask permission from your partner? / *Ngati mukufuna kupita kuchipatala, kodi mumayenera kuti mupemphe chilolezo kwa amuna anu?*

**Dimension of Autonomy IV: Discretion Over Earned Income**

Goal: To determine the extent of the participant’s involvement in decisions relating to access to family income

Control over **access to family income related to pregnancy and birth**

- Who usually decides how your husband’s earnings will be used: you, your partner, you and your partner jointly, or someone else? / *Kodi kawirikawiri ndi ndani amene amaganiza za, mmene ndalama imene amuna anu amapeza igwiritsidwire ntchito, inu, amuna anu kapena inu ndi amuna anu limodzi?*
- Who usually decides how the money you earn will be used: you, your partner, you and your partner jointly, or someone else? / *Kodi kawirikawiri ndi ndani amene amaganiza za, mmene mungagwiritsile ntchito ndalama yanu imene inu mwapeza; inu, amuna anu, kapena inu ndi amuna a nu limodzi?*
- If you wanted to use your family’s money to pay for medical treatment or transportation to a health facility, would you be able to do so? / *Mutakhala kuti mukufuna kugwiritsa ntchito ndalama ya pakhomo panu pofuna kulipira kuchipatala kapena kuyendera kupita kuchipatala, kodi mungathe kutero?*
